# Overview of Molecular Diagnostics in Irish Clinical Oncology

**DOI:** 10.12688/hrbopenres.13822.1

**Published:** 2024-03-26

**Authors:** Tyler Medina, Seán O. Hynes, Maeve Lowery, Patrick Gillespie, Walter Kolch, Cathal Seoighe

**Affiliations:** 1 School of Mathematical & Statistical Sciences, University of Galway, Galway, County Galway, Ireland; 2 SFI Centre for Research Training in Genomics Data Science, Science Foundation Ireland, Dublin, Ireland; 3 Discipline of Pathology, School of Medicine, University of Galway, Galway, County Galway, Ireland; 4 Division of Anatomical Pathology, University Hospital Galway, Galway, Ireland; 5 Trinity St James’s Cancer Institute, St James's Hospital and Trinity College Dublin, Dublin, Ireland; 6 CURAM, SFI Research Centre for Medical Devices, University of Galway, Galway, County Galway, Ireland; 7 Health Economics and Policy Analysis Centre, Institute for Lifecourse and Society, University of Galway, Galway, County Galway, Ireland; 8 Conway Institute of Biomolecular and Biomedical Research, University College Dublin, Dublin, Ireland; 9 Systems Biology Ireland, University College Dublin, Dublin, Ireland

**Keywords:** personalised medicine, molecular diagnostics, genomics, cancer, clinical oncology, Ireland

## Abstract

**Background:**

Molecular diagnostics are critical for informing cancer patient care. In Ireland, the National Cancer Control Programme (NCCP) develops cancer therapy regimens, which include relevant information on molecular indications. Here, we present a collated overview of the current molecular indications of all NCCP systemic anti-cancer therapy regimens and the funding statuses of their associated drugs. Furthermore, we also provide estimates for the scale of required molecular testing in cancer therapy and for the clinical genetic sequencing capacity of Ireland, and provide a summary of current cancer clinical trials in Ireland which have molecular components.

**Methods:**

Through a combination of web scraping, keyword search, and manual review, we performed a full review of all 757 indications included in the 476 therapy regimens published to date by the NCCP to identify therapy indications with explicit molecular criteria. For all cancer types identified in these indications, we obtained incidence rates in Ireland from National Cancer Registry Ireland to predict the number of patients yearly who stand to benefit from a molecular test. We then applied molecular subtype rates from published literature to estimate the number of patients who would then qualify for a relevant molecularly guided therapy.

**Results:**

We identified 210 indications for 148 NCCP therapy regimens that include molecular criteria. These 210 molecular indications encompassed 85 genetic criteria, 137 cellular biomarker criteria, 57 molecularly informed drugs, and over 20 cancer types. We estimated that up to approximately 50% of cancer patients in Ireland could qualify for a molecular test and that the majority of tested patients would qualify for a treatment informed by a molecular test.

**Conclusions:**

As personalised cancer medicine continues to develop in Ireland, this study will provide a baseline understanding of current practices. We anticipate that work such as this will help to inform planning in the healthcare system.

## Introduction

Modern genetics and genomics have played a vital role in human health for decades. However, since the advent of high-throughput next-generation sequencing (NGS), the role of genomics and molecular diagnostics in healthcare has increased dramatically ^1^. As the science, engineering, and data analysis surrounding genomics continue to develop through research and innovation, genomics technologies progressively move from research and development into practical clinical usage in applications ranging from neonatal screening ^2^ and hereditary disease risk ^3^ to chemotherapy management and prognostics ^4^.

To facilitate the integration of genomics and healthcare, many nations are in the process of developing or implementing strategies, legislation, policy, and infrastructure for clinical genomics ^5– 9^. Ireland is among these nations, having recently published a national plan for genomics medicine under the National Genomics and Genetics Strategy, which will oversee and guide implementation of the strategy as part of the national healthcare system in coming years ^10^.

While science and innovation drive novel technologies and techniques in genomics, familiarity with current clinical practices is vital to matching research effort and expertise to clinical need and application. Here we aim to highlight actionable and informative molecular diagnostics in use in clinical oncology in Ireland by examining the cancer therapies and clinical trials currently informed by molecular diagnostics in Ireland. In addition, amidst increasing cancer incidence each year, we predict the number of patients in Ireland requiring a molecular diagnostic yearly and the number that would potentially benefit from molecular diagnostics and compare this to the availability of NGS infrastructure in major hospitals around the country.

## Molecular Diagnostics in Cancer Treatment Regimens in Ireland

Under the Health Service Executive (HSE), the National Cancer Control Programme (NCCP) is the leading national body addressing the diagnosis and treatment of cancer in Ireland. With the principal aim of implementing the Irish National Cancer Strategy, the NCCP's activities include reviewing new cancer therapies and developing national regimens for their use as part of the National Cancer Information System ^11^.

New cancer drugs approved by the European Medicines Agency are assessed by the National Centre for Pharmacoeconomics, Ireland (NCPE) to produce a health technology assessment (HTA), which addresses the benefit vs. financial cost of the drug in question and recommends whether the drug should or should not be reimbursed by the HSE ^12^. These reports, as well as information from experts and research, are assessed by the NCCP Technology Review Committee to recommend cancer drugs for funding under HSE drug schemes such as the Oncology Drugs Management Scheme (ODMS) or the Primary Care Reimbursement Services (PCRS) community drugs schemes ^13, 14^.

Independent of the funding status of a drug, the NCCP also develops, manages, and reviews national drug regimens addressing when and how these drugs should be used. In addition to information about drug combinations and dosing, these regimens also include, when relevant, the molecular indications required for the use of certain drugs in particular cancer types ^15^. Note that while these regimens set guidelines for therapy, they are not exhaustive and clinical practice may differ when appropriate.

### Genetic Indications

Cancer is, by nature, a disease of genetic origins ^16^. Though there are many different genome sequence mutations associated with many cancer types, only a small subset of these are currently known to be clinically informative or actionable, typically by informing diagnosis, prognosis, and/or treatment options ^17^. For example, the *EGFR* gene encodes a tyrosine kinase which, when activated, signals for increased DNA replication and general cell proliferation; as such, over-activation of EGFR is associated with a variety of cancer types, including non-small cell lung cancer (NSCLC), in which approximately 14% of European patients harbour an EGFR-activating mutation ^18^. For these patients, tyrosine-kinase inhibitor (TKI) therapies specifically targeting EGFR (e.g., osimertinib, gefitinib) are more effective and are associated with more favourable outcomes compared to chemotherapy ^19, 20^. In colorectal cancer patients, however, the presence of KRAS-activating mutations greatly reduces the efficacy of anti-EGFR TKI chemotherapies, as KRAS is a downstream activation target of the EGFR signalling pathway; once permanently activated through mutation, KRAS promotes tumour growth regardless of EGFR inhibition, and is associated with poorer outcomes ^21^.

As in these examples, identifying genetic mutations can be critical in directing cancer treatment. Among all cancer treatment regimens developed by the NCCP, there are currently 13 genetic factors (including 2 broader genetic phenotypes) informing 85 therapy indications across 63 chemotherapy regimens. These regimens involve combinations of 42 different genomics-informed drugs, 37 of which are approved for funding through either the PCRS or the ODMS for approved indications ( Table 1) ^15, 22– 25^.

**Table 1.** Aggregate summary of genetic indications in NCCP cancer therapies. Genetic indications per cancer type are listed with their associated drugs and drug reimbursement status in Ireland. (NSCLC: non-small cell lung cancer; mCRC: metastatic colorectal cancer; mCRPC: metastatic castration-resistant prostate cancer; ALL: acute lymphoblastic leukaemia; CLL: chronic lymphocytic leukaemia; AML: acute myeloid leukaemia; CML: chronic myelogenous leukaemia; GIST: gastrointestinal stromal tumour; PCRS: Primary Care Reimbursement Service; ODMS: Oncology Drugs Management System; MSI-H: microsatellite instability-high; dMMR: deficient mismatch repair; HRD: homologous recombination deficiency).

Cancer Type	Subtype	Indication	Drugs	Reimbursement
Breast	metastatic breast cancer	*BRCA1/2* germline mutation	talazoparib	PCRS
Lung	NSCLC	*ALK* mutation	alectinib	PCRS
			brigatinib	PCRS
			ceritinib	PCRS
			crizotinib	PCRS
			lorlatinib	PCRS
		EGFR-activating mutation	afatinib	PCRS
			dacomitinib	PCRS
			erlotinib	PCRS
			erlotinib and bevacizumab	erlotinib: PCRS; bevacizumab: Hospital
			gefitinib	PCRS
			osimertinib	PCRS
		*EGFR* T790M mutation	osimertinib	PCRS
		normal *EGFR* and *ALK*	atezolizumab	ODMS
			ipilimumab and nivolumab	ODMS
			pembrolizumab	ODMS
		*ROS1* mutation	crizotinib	Reimbursement for indication not approved
			entrectinib	PCRS
Gastro-intestinal	mCRC	normal *RAS*	cetuximab	Hospital
			panitumumab	Hospital
		MSI-H or dMMR	pembrolizumab	ODMS
			ipilimumab and nivolumab	ODMS
Skin	metastatic melanoma	*BRAF* V600 mutation	dabrafenib	PCRS
			dabrafenib and trametinib	PCRS
			encorafenib and binimetinib	PCRS
			vemurafenib	PCRS
			vemurafenib and cobimetinib	PCRS
Gynae-cological	epithelial ovarian, fallopian tube, and peritoneal cancers	HRD+, *BRCA1/2* somatic mutation, or genomic instability	olaparib and bevacizumab	olaparib: ODMS; bevacizumab: Hospital
		*BRCA1/2* germline or somatic mutation	olaparib	PCRS
Genito-urinary	mCRPC	*BRCA1/2* germline or somatic mutation	olaparib	PCRS
			niraparib and abiraterone acetate (akeega®)	PCRS
Leukaemia	ALL	*BRC-ABL1* fusion	inotuzumab ozogamicin	ODMS
		*BRC-ABL1* fusion with T315I mutation	ponatinib	PCRS
		*BRC-ABL1* fusion negative	blinatumomab	ODMS
	CLL	*TP53* mutation or deletion	acalabrutinib	PCRS
			idelalisib and rituximab	idelalisib: PCRS; rituximab: Hospital
			ibrutinib	PCRS
			venetoclax	PCRS
			zanubrutinib	PCRS
	AML	*FLT3* mutation	midostaurin	PCRS
	CML	*BRC-ABL1* fusion	bosutinib	PCRS
			asciminib	PCRS
		*BRC-ABL1* fusion with T315I mutation	ponatinib	PCRS
Sarcoma	GIST	*CD117* mutation	imatinib	PCRS
Tumour-agnostic		*NTRK* fusion	larotrectinib	PCRS

### Techniques and Technologies

Depending on clinical purpose and cost, testing for relevant genetic mutations in cancer occurs at several levels of scale. Small-scale single-gene tests can be used to identify known point mutations, such as *EGFR* T790M or *KRAS* G12C chemotherapy resistance mutations ^26, 27^, or to identify known fusion genes, such as the *BCR-ABL1* gene fusion found in chronic myelogenous leukaemia (CML) ^28^. These single-gene tests are generally performed using techniques such as quantitative polymerase chain reaction (qPCR) or fluorescent in-situ hybridization (FISH), and can also be performed on both Sanger sequencing and next-generation sequencing (NGS) platforms, though using high-throughput NGS with very small targets is generally not cost efficient without very large numbers of samples.

Multiple genes can be tested for mutations simultaneously by sequencing on an NGS instrument. These NGS assays range from small disease-focused gene panels, targeting tens to hundreds of genes; to whole exome sequencing (WES or WXS), targeting tens of thousands of genes; to whole genome sequencing (WGS), which generates data from both genic and non-genic regions. In all NGS applications, results can then be subset virtually to focus on disease-specific genes or regions of interest. While methods like qPCR or genotyping microarrays can be used to detect known mutations, genome sequencing does not require *a priori* knowledge of mutations of interest, thus allowing for discovery of novel relevant genomic variation in cancer ^29^. While novel mutations are not likely to be clinically actionable upon discovery, they may have potential for use in research, trials, and treatment in the future. More comprehensive genomic sequencing additionally allows for more complex genomic profiling strategies which can further inform disease aetiology, progression, and prognosis.

In Ireland, qPCR and FISH single-gene tests, gene panels including ThermoFisher's Oncomine Focus panels and other ThermoFisher Ion AmpliSeq small gene panels, and clinical exome gene panels are all routinely used. While whole genome sequencing can be clinically useful, this is generally not performed in Ireland as routine care outside of clinical trials or research applications.

In addition to the genetic sequencing performed by Irish medical laboratories, patient samples are also sent to external sequencing facilities in cases requiring, for example, rapid turnaround time, Sanger sequencing variant confirmation, or specialty assay sequencing. Notably, homologous recombination deficiency (HRD) has recently been added as an NCCP indication for olaparib treatment of ovarian cancer. While largely determined by the presence of deleterious *BRCA1/2* mutations, HRD is a wider genetic phenotype influenced by larger genomic factors such as loss-of-heterozygosity and rearrangement events. Similarly, high microsatellite instability (MSI-H), which was recently added as an NCCP indication for immune checkpoint inhibitors in colorectal cancer, requires profiling of multiple locations throughout the genome. Both HRD and MSI-H testing thus require larger or specialty NGS gene panels, such as the Myriad MyChoice HRD test, FoundationOne panel, and Illumina TSO500 panel, all of which are currently being considered for use in Ireland. These external tests are generally funded under hospital departmental budgets rather than being reimbursed directly by the HSE, though efforts are underway by several hospitals to develop the infrastructure required to perform more genetic tests domestically in public facilities.

### Cellular Biomarker-Based Indications

In addition to identifying mutated cancer-associated genes, confirming the presence of cellular biomarkers, which commonly include hormone receptors and antigens involved in immune cell recognition, can also be vital for accurate cancer diagnosis and treatment decisions. Lymphoma subtypes, for example, each exhibit characteristic immunophenotypes which can be essential for differential diagnosis of cancers that are otherwise morphologically similar ^30, 31^.

Biomarkers expressed on the cell surface can also serve as key drug targets. Antibody-based therapies target only specific cell types exhibiting the target antigen, and thus can activate or inhibit cellular signalling pathways or elicit a patient immune response against target cells, while limiting the potential deleterious effects of cancer treatment. Antibody-drug conjugates, such as brentuximab vedotin, further exploit this specificity by directing otherwise highly toxic chemotherapy drugs only to cells exhibiting the target antigen ^32^.

Complementary to antibody-based therapies, small molecule drugs can also reach intracellular targets. For example, several treatment routes exist to reduce the growth-promoting effect of oestrogen on oestrogen-receptor-positive (ER+) breast tumours, including anastrozole, which binds aromatase enzymes to inhibit the production of oestrogen in the body; tamoxifen, which inhibits oestrogen binding by blocking oestrogen receptors; and fulvestrant, which binds and destabilises oestrogen receptors, inducing their breakdown by the cell ^33^.

Like genetic mutations, the presence or absence of cellular biomarkers can play a critical role in diagnosis, prognosis, and treatment of a patient. In Ireland, the presence or absence of 10 markers are a factor for 137 indications for 22 different therapies across 98 treatment regimens published by the NCCP, and are of particular importance for informing breast cancer and lymphoma treatments, which account for 75% of these indications. Of the 22 included therapies, 19 have funding through the ODMS and PCRS ( Table 2) ^15, 22– 25^.

**Table 2.** Aggregate summary of cellular biomarker-based indications in NCCP cancer therapies. Indications are listed per cancer type with their associated drugs and drug reimbursement status in Ireland. (NSCLC: non-small cell lung cancer; mCRC: metastatic colorectal cancer; GEJ: gastro-oesophageal junction; HNSCC: head and neck squamous cell carcinoma; B-ALL: B-cell acute lymphoblastic leukaemia; AML: acute myeloid leukaemia; NHL: non-Hodgkin lymphoma; PCRS: Primary Care Reimbursement Service; ODMS: Oncology Drugs Management System).

Cancer Type	Subtype	Indication	Drugs	Reimbursement
Breast		ER+	fulvestrant	PCRS
			tamoxifen	PCRS
		HR+	anastrozole	PCRS
			exemestane	PCRS
			letrozole	PCRS
		HER2+	trastuzumab	Hospital
			trastuzumab and pertuzumab	trastuzumab: Hospital; pertuzumab: ODMS
			trastuzumab/ pertuzumab (Phesgo ^®^)	ODMS
			trastuzumab emtansine (Kadcyla ^®^)	ODMS
			neratinib	PCRS
			lapatinib	PCRS
		HER2-, HR+	exemestane	PCRS
			aromatase inhibitor or fulvestrant	PCRS
		HER2-, HR-, PD-L1+	atezolizumab	ODMS
Lung	NSCLC	PD-L1+	atezolizumab	ODMS
			durvalumab	ODMS
			pembrolizumab	ODMS
Gastro-intestinal	mCRC	EGFR+	cetuximab	Hospital
	metastatic stomach adenocarcinoma	HER2+	trastuzumab	Hospital
	metastatic gastric or GEJ cancer	HER2+	trastuzumab	Hospital
	GEJ adenocarcinoma	HER2-, PD-L1+	pembrolizumab	ODMS
	oesophageal carcinoma	PD-L1+	pembrolizumab	ODMS
	oesophageal squamous cell carcinoma	PD-L1+	nivolumab	ODMS
Genito-urinary	urothelial carcinoma	PD-L1+	atezolizumab	ODMS
			pembrolizumab	ODMS
Gynae-cological	cervical cancer	PD-L1+	pembrolizumab	Reimbursement by exception
Head & Neck	HNSCC	PD-L1+	pembrolizumab	ODMS
Leukaemia	B-ALL	CD19+	blinatumomab	ODMS
		CD22+	inotuzumab ozogamicin	ODMS
	AML	CD33+	gemtuzumab ozogamicin	ODMS
Lymphoma	Hodgkin lymphoma	CD30+	brentuximab vedotin	ODMS
		CD20+	rituximab	Hospital
	non-Hodgkin B-cell lymphomas	CD20+	rituximab	Hospital
	follicular lymphoma	CD20+	rituximab	Hospital
		CD20+	obinutuzumab	ODMS
	systemic anaplastic large cell lymphoma	CD30+	brentuximab vedotin	ODMS
	cutaneous T-cell lymphoma	CD30+	brentuximab vedotin	ODMS

### Techniques and Technologies

The presence of cellular biomarkers can be determined either by direct detection, or by some indirect indication of their presence. Immunohistochemistry (IHC) techniques, which remain the gold standard for direct determination, use a combination of an antigen-specific antibody and a dye or fluorophore to indicate antigen presence in cancer tissue samples via microscopy ^34^. While this technique can generally only detect one antigen per assay, the process can be parallelized in appropriate tissue samples via flow cytometry, such that multiple antibodies can be applied, allowing several antigens to be detected on cancer cells simultaneously ^35, 36^.

Indirect detection, instead, can be accomplished through gene expression analysis. Rather than detecting an antigen of interest via an antibody, this approach involves the quantification of RNA transcripts encoding the biomarker. Techniques for measuring expression analysis are similar to those for detecting DNA mutations and include reverse transcription qPCR (RT-qPCR), expression microarrays, and next-generation RNA sequencing (RNA-seq).

These methods also scale similarly to DNA mutation detection methods: qPCR is limited to measuring the expression of single genes, while microarrays and RNA-seq are able to simultaneously quantify thousands of transcripts. Of particular note is that RNA-seq, in addition to expression analysis, also allows for mutation detection by default. This includes more complex mutations such as fusion genes, which are frequently highly associated with cancer and can serve as drug targets for inhibitors such as ponatinib, which inhibits BCR-ABL1 fusion proteins found in CML ^37^, and larotrectinib, a novel tumour-agnostic Trk inhibitor that can be used for any cancer in which *NTRK*-family fusions are detected ^38^. While RNA expression assays are less commonly used in clinical practice, recent studies have shown comparable test results between RNA-seq and IHC ^39^.

In Irish hospitals, cellular biomarker detection methods generally include single gene tests like IHC and RT-qPCR. In addition, the external testing service Oncotype DX ^®^ is available in Ireland for breast cancer patients and uses RT-qPCR to measure the expression of 16 genes, including *HER2* and both oestrogen and progesterone receptor genes ^40– 42^. While RNA-seq remains uncommon, reimbursement for larotrectinib in Ireland notably requires submission of RNA-seq results ^43^.

## Requirement for Cancer Molecular Diagnostics

**Figure 1.**
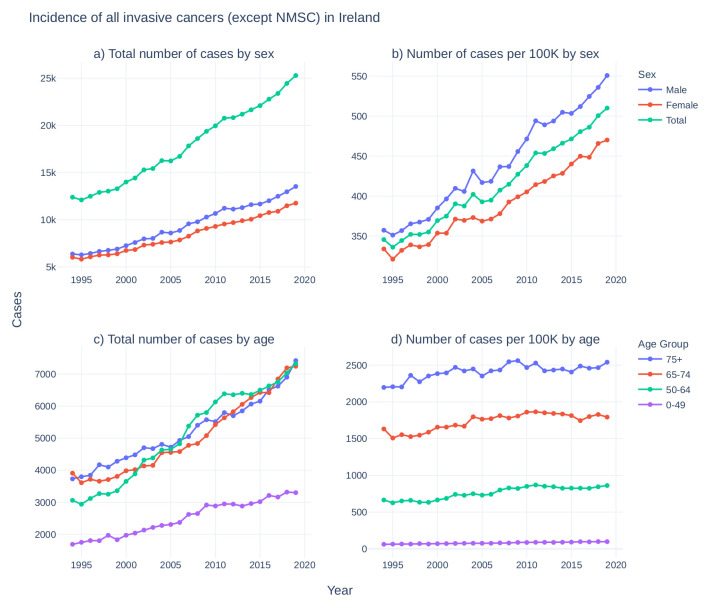
Incidence of all invasive cancers (except NMSC) in Ireland from 1994–2019. **Top:** For males (blue), females (red), and total population (green), **a**) case counts of cancers per year and **b**) case counts per 100,000 individuals in each category per year. **Bottom:** For 0–49 (purple), 50–64 (green), 65–74 (red), and 75+ (blue) years old, **c**) cancer case counts per year and **d**) cancer case counts per 100,000 individuals in each category per year. Cancer incidence data taken from National Cancer Registry Ireland ^44^. National population estimates per year taken from the Central Statistics Office Ireland ^45^. (NMSC: non-melanoma skin cancer).

**Figure 2.**
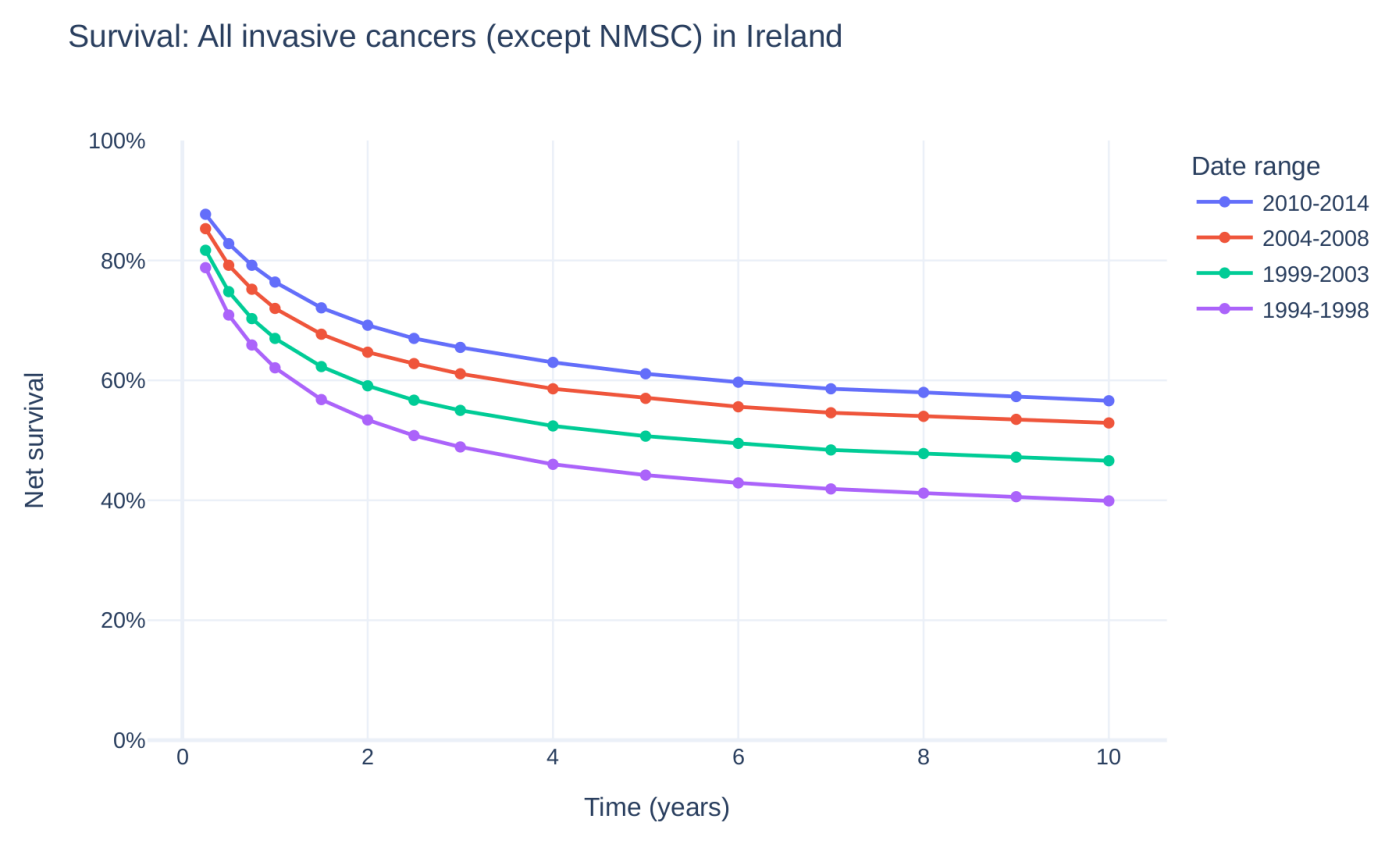
Net survival of all invasive cancers (except NMSC) in Ireland over time, from 1994–2014. Survival curve estimates showing percent net survival at selected time points for diagnoses made during the given time period. Data provided by and plot adapted from National Cancer Registry Ireland ^44^. (NMSC: non-melanoma skin cancer).

Data on the incidence of cancer in Ireland has been centrally recorded by National Cancer Registry Ireland (NCRI) since 1994. While the total incidence of cancer in Ireland has doubled since 1994 ( Figure 1a, 1c), the rate of cancer incidence has increased by approximately 50% ( Figure 1b) and age-adjusted incidence has increased by approximately 15% ( Figure 1d), reflecting at least in part the advancing age profile of the larger population and increases in life expectancy ^44, 45^. Latest available figures (from 2020) show a current 1 in 2 lifetime risk of invasive cancer ^46^. Fortunately, overall cancer survival in Ireland has also increased ( Figure 2), with a gain of approximately 15 percentage point survivorship over the same time period ^47^.

Advances in personalised medicine continue to contribute to this survival improvement, with cancer molecular diagnostics enabling a wide range of modern therapy options. However, it is not clear how many patients in Ireland currently receive or stand to benefit from molecular cancer diagnostics. Data on the rate of molecular diagnostics usage in Ireland is not publicly available and is not currently centrally recorded. This information is relevant to quantification of the potential benefits of genomic tests on the population level and for resource planning, not least as part of the National Genomics and Genetics Strategy. Furthermore, while NCRI collects and provides information on cancer incidence, specific cancer rates are categorised by International Classification of Diseases 10th revision (ICD-10) definitions largely classed by tissue type, leaving molecular subtype rates in Ireland unknown.

Based on current disease-informing molecular diagnostics listed in NCCP treatment regimens, cancer incidence rates published by NCRI, published studies on molecular cancer subtypes, and the single most common molecular subtype per cancer, we estimate that over 12,000 patients should be receiving some form of molecular diagnostic test yearly to identify the subpopulation of at least 7,000 cancer patients that stand to benefit from current molecular-diagnostic-guided therapeutics used in Ireland. These include over 1,500 patients who would qualify for a genetic-guided therapy, and 6,500 patients who would qualify for a cellular biomarker-guided therapy ( Table 3 and Table 4). This testing burden represents approximately 50% of the 24,000 invasive cancer cases in Ireland yearly (excluding non-melanoma skin cancer), with about 30% directly benefiting from a test result ^46^. It should be noted that these numbers only include tests that directly inform therapy, and do not include the large body of molecular tests performed primarily for diagnostic or prognostic purposes. In addition, *NTRK*-fusion testing is not included in these estimates, as treatment is tumour-agnostic and *NTRK*-fusion incidence is quite variable across various tumour types ^48^.

**Table 3.** Predicted incidence of cancers with genetic indications for therapy in Ireland. Yearly incidence of cancer types with a genetic indication in Ireland, with predicted numbers of positive molecular diagnoses based on rates from literature. Incidence in Ireland published by National Cancer Registry Ireland ^46^, unless otherwise noted by citation. Molecular subtype rate estimates (MD+ Rate) for each cancer obtained from indicated references. (ICD-10: International Classification of Diseases 10th Revision; MD: Molecular diagnostic; NSCLC: non-small cell lung carcinoma; mCRC: metastatic colorectal cancer; B-ALL: B-cell acute lymphoblastic leukaemia; CLL: chronic lymphocytic leukaemia; AML: acute myeloid leukaemia; CML: chronic myelogenous leukaemia; mCRPC: metastatic castration-resistant prostate cancer; GIST: gastrointestinal stromal tumour; MSI-H: microsatellite instability-high; dMMR: deficient mismatch repair) *Only female cases included. **Chemotherapy resistance mutation incidence is variable, with incidence typically increasing in response to therapy.

Cancer Type	ICD-10 Label	Subtype	Incidence in Ireland	Molecular Diagnostic (MD)	MD+ Rate	MD+ Incidence in Ireland	References
Breast	C50: Malignant neoplasm of breast		3363 *	*BRCA1/2* germline mutation	0.024	81	51– 54
Lung	C34: Malignant neoplasm of bronchus and lung	NSCLC ^55– 57^	2268	*ALK* mutation	0.05	113	58– 60
				*ROS1* mutation	0.02	45	61
				EGFR-activating mutation	0.14	320	18, 58
				*EGFR* T790M mutation	Builds **		62, 63
Gastro-intestinal	C18-21: Malignant neoplasm of: - colon - rectosigmoid junction - rectum - anus and anal canal	mCRC ^64^	1058	normal *RAS*	0.39	416	64
				MSI-H or dMMR	0.04	42	65– 68
Skin	C43: Malignant melanoma of skin	Melanoma	1170	*BRAF* V600 mutation	0.5	585	69– 71
Leukaemia	C91.0: Acute lymphoblastic leukaemia [ALL]	B-ALL ^72^	49	*BCR-ABL1* fusion	0.04 paediatric, 0.25 adult	5	73
				*BCR-ABL1* fusion with T315I mutation	Builds **		74
	C91.1: Chronic lymphocytic leukaemia of B-cell type	CLL	202	*TP53* mutation or deletion	0.1	20	75
	C92.0: Acute myeloblastic leukaemia [AML]	AML	138	*FLT3* mutation	0.3	41	76
	C92.1: Chronic myeloid leukaemia [CML], BCR/ABL-positive	CML	63	*BCR-ABL1* fusion	0.94	59	77
				*BCR-ABL1* fusion with T315I mutation	Builds **		77
Gynae-cological	C56: Malignant neoplasm of ovary	Epithelial ovarian cancer ^78– 80^	360.9	*BRCA1/2* germline or somatic mutation	0.25	90	81– 83
	C57.0: Malignant neoplasm: Fallopian tube	Fallopian tube cancer	25		0.35	9	81– 83
	C48: Malignant neoplasm of retroperitoneum and peritoneum	Peritoneal carcinoma	24 *		0.16	4	81– 83
Genito-urinary	C61: Malignant neoplasm of prostate	mCRPC ^84, 85^	534	*BRCA1/2* germline or somatic mutation	0.14	75	86
Sarcoma	C49: Malignant neoplasm of other connective and soft tissue	GIST ^87^	20	*CD117* mutation	0.8	16	88, 89
**Total, max per cancer type**			9274			1721	

**Table 4.** Predicted incidence of cancers with cellular biomarker indications for therapy in Ireland. Yearly incidence of cancer types with a cellular biomarker-based diagnostic in Ireland, with predicted numbers of positive molecular diagnoses based on rates from literature. Incidence in Ireland published by National Cancer Registry Ireland ^46^, unless otherwise noted by citation. Molecular subtype rate estimates (MD+ Rate) for each cancer obtained from indicated references. (ICD-10: International Classification of Diseases 10th Revision; MD: Molecular diagnostic; NSCLC: non-small cell lung carcinoma; mCRC: metastatic colorectal cancer; GEJ: gastro-oesophageal junction; ALCL: anaplastic large cell lymphoma; CTCL: cutaneous T-cell lymphoma; HNSCC: head and neck squamous cell carcinoma; B-ALL: B-cell acute lymphoblastic lymphoma; AML: acute myeloid leukaemia) *Only female cases included.

Cancer Type	ICD-10 Label	Subtype	Incidence in Ireland	Molecular Diagnostic (MD)	MD+ Rate	MD+ Incidence in Ireland	References
Breast	C50: Malignant neoplasm of breast		3363 *	ER+	0.806	2711	90
				HR+	0.818	2751	90
				HER2+	0.154	517	90
				PD-L1+	0.197	663	91
Lung	C34: Malignant neoplasm of bronchus and lung	NSCLC ^55– 57^	2268	PD-L1+	0.336	739	92, 93
Gastro-intestinal	C18-21: Malignant neoplasm of: - colon - rectosigmoid junction - rectum - anus and anal canal	mCRC ^64^	1058	EGFR+	0.6	635	94, 95
	C16: Malignant neoplasm of stomach	stomach or GEJ cancer	557	HER2+	0.221	123	96
	C15: Malignant neoplasm of oesophagus	oesophageal or GEJ cancer	515	PD-L1+	0.45	232	97– 100
Lymphoma	C81: Hodgkin lymphoma	Hodgkin lymphoma ^101^	127	CD30+	1.00	127	30, 101
	C82: Follicular lymphoma C83: Non-follicular lymphoma C85: Other and unspecified types of non-Hodgkin lymphoma C88: Malignant immunoproliferative diseases	non-Hodgkin B-cell lymphomas	712	CD20+	0.98	698	102
	C84: Mature T/NK-cell lymphomas	ALCL and CTCL	83	CD30+	1.00	83	103
Head & Neck	C00-14: Malignant neoplasms of lip, oral cavity and pharynx C30-C32: Malignant neoplasm of: - nasal cavity and middle ear - accessory sinuses - larynx	HNSCC	786	PD-L1+	0.85	668	104
Genito- urinary	C65-68: Malignant neoplasm of: - renal pelvis - ureter - bladder - other and unspecified urinary organs	urothelial carcinoma ^105– 107^	536	PD-L1+	0.303	162	108, 109
Gynae-cological	C53: Malignant neoplasm of cervix uteri	cervical cancer	253	PD-L1+	0.85	215	110, 111
Leukaemia	C91.0: Acute lymphoblastic leukaemia [ALL]	B-ALL ^72^	49	CD19+	1.00	49	112
				CD22+	0.98	48	113
	C92.0: Acute myeloblastic leukaemia [AML]	AML	138	CD33+	0.85	117	114
**Total, max per cancer type**			8682			6599	

To accommodate the clinical needs of these individuals, clinical laboratories in the Republic of Ireland operate several makes of instruments, each with their own capacity and throughput. In total, there are four Illumina NextSeq, one Illumina MiniSeq, and four ThermoFisher Ion Torrent NGS instruments currently operating in clinical practice across 5 Irish hospitals. In addition, there are a number of qPCR machines available for single-gene tests, as well as one Sanger sequencing platform for confirmation testing. Based on published technical specifications ^49, 50^, the combination of high-throughput NextSeq and Ion Torrent instruments in Ireland represent a maximum nominal capacity of approximately 44 – 68 deep whole exomes sequenced in a 30 hour period, depending on targeted depth, exome size, and amplification, though in reality this number is also greatly dependent upon sample batching, laboratory operation and sample preparation time, specific instrument configuration, and operating costs, among numerous other factors.

## Molecular Indications for Clinical Trial Inclusion

In addition to routine care pathways, clinical trials offer some patients access to cancer therapies that would not otherwise be available, typically because the therapy is novel or is not yet offered in Ireland. Clinical trials for cancer therapies dictate strict enrolment criteria, and these are often based on molecular diagnosis of cancer subtypes. Cancer Trials Ireland, for example, currently lists 86 clinical trials for cancer available in the country. Of these, at least 43 list a molecular diagnostic as either eligibility criteria or as a factor in the trial ( Table 5) ^115^. For example, the KRYSTAL-10 and LOXO 101 trials are both currently active in Ireland: KRYSTAL-10 is currently recruiting at several Irish hospitals, and is investigating the use of a novel KRAS-inhibiting drug, known currently as MRTX849, to treat colorectal cancer patients who have the KRAS-activating G12C mutation ^116^, while LOXO 101 is investigating the use of larotrectinib to treat any cancer harbouring an *NTRK* gene fusion that has been confirmed via molecular assay ^117^. Besides drug trials, other efforts in the field of genomics are also underway in clinical trials in Ireland, including fundamental research into the genetic profiling of cancers and DNA biobanking ^118^.

**Table 5.** Current cancer clinical trials in Ireland with a molecular component. Summary of cancer clinical trials listed by Cancer Trials Ireland whose study designs include a molecular component. Clinical trial IDs are given as clinicaltrials.gov IDs where available (except trial ITCC-059, which is listed by EudraCT ID). (miRNA: microRNA; GEJ: gastro-oesophageal junction; MIBC: muscle invasive bladder cancer; ctDNA: circulating tumour DNA; dMMR: deficient mismatch-repair; HNSCC: head and neck squamous cell carcinoma; AML: acute myeloid leukaemia; MDS-EB2: myelodisplastic syndromes with excess blasts-2; NSCLC: non-small cell lung carcinoma; NGS: next-generation sequencing; DLBCL: diffuse large B-cell lymphoma; CML: chronic myelogenous leukaemia; B-ALL: B-cell acute lymphoblastic leukaemia; CNS: central nervous system; ALL: acute lymphoblastic leukaemia; MDS: myelodysplastic syndrome; JNML: juvenile myelomonocytic leukaemia; HRRm: homologous recombination repair mutation; HRD: homologous recombination deficiency).

Cancer Type	Subtype	Trial Name	Clinical Trial ID	Molecular Component
Breast		SHAMROCK study	NCT05710666	Requires HER2+
		DESTINY-Breast05	NCT04622319	Requires HER2+
		SASCIA	NCT04595565	Requires HER2-
		KEYNOTE-B49	NCT04895358	Requires HER2-, HR+
		EPIK-B5	NCT05038735	Requires HER2-, HR+, *PIK3CA* mutation
		Proteomics/ Molecular Breast	NCT01840293	Gene-protein interaction study
CNS	glioma	Serum Protein Markers for Glioma	NCT03698201	Identification of blood miRNA biomarkers
Gastro-intestinal	gastric cancer	FORTITUDE-101	NCT05052801	Requires FGFR2b overexpression; excludes HER2+
	colorectal cancer	KRYSTAL-10	NCT04793958	Requires *KRAS* G12C mutation
	stomach and oesophageal cancers	HERIZON-GEA-01 (ZWI-ZW25-301) Zymeworks	NCT05152147	Requires HER2+
	gastric or GEJ adenocarcinoma	DESTINY DS8201-A-U306	NCT04704934	Requires HER2+
	pancreatic adenocarcinoma	Astellas 8951-CL-5201	NCT03816163	Requires CLDN18.2+
Genito-urinary	urothelial carcinoma / MIBC	MK3475-905 (KEYNOTE-905)	NCT03924895	Requires tissue for PD-L1 testing
	MIBC	IMvigor011 B042843	NCT04660344	Requires ctDNA positive; will perform PD-L1 expression testing
Gynae-cological	endometrial carcinoma	ENGOT-en15/ KEYNOTE-C93-00/ GOG-3064	NCT05173987	Requires dMMR
Head & Neck	HNSCC	MK-3475-630/ KEYNOTE-630	NCT03833167	Requires tissue for PD-L1 testing
	HNSCC	MK-3475-689	NCT03765918	Stratified by PD-L1 expression
Leukaemia	AML or MDS-EB2	HOVON 156	NCT04027309	Requires *FLT3* mutation
		HOVON 150	NCT03839771	Requires *IDH1/2* mutation
Lung	NSCLC	22-09 ADEPPT	NCT05673187	Requires *KRAS* G12C mutation
		KRYSTAL-12	NCT04685135	Requires *KRAS* G12C mutation
		KRYSTAL-7	NCT04613596	Requires *KRAS* G12C mutation; phase depends on PD-L1 expression
		AcceleRET-Lung	NCT04222972	Requires *RET* fusion; excludes other known driver mutations such as *EGFR*, *ALK*, *ROS-1*, *MET*, and *BRAF* mutations
		AbbVie M14-239	NCT03539536	Requires c-Met overexpression; excludes *EGFR* mutation
		CA224-104 (RELATIVITY)	NCT04623775	Excludes *EGFR*, *ALK*, *ROS-1*, and *BRAF* V600E mutations
		23-12 LATIFY	NCT05450692	Excludes *EGFR* and *ALK* mutations
		22-15 PLAN	NCT05542485	ctDNA genotyping via NGS
		22-23 NeoCOAST-2	NCT05061550	Will confirm PD-L1, *ALK*, and *EGFR* status
Lymphoma	DLBCL	MOR208C310	NCT04824092	Requires CD20+
Paediatric	CML	ITCC-054	NCT04258943	Requires *BCR-ABL1* fusion; excludes *BCR-ABL1* T315I or V299L mutations
	B-ALL	ITCC-059	2016-000227-71 (EudraCT)	Requires CD22+
	CNS tumour	LOXO TRK 15003	NCT02637687	Requires *NTRK* fusion
	ALL or biphenotypic leukaemia	Interfant 06	NCT00550992	Requires *MLL* rearrangement; excludes *BCR-ABL1* fusions and t(8;14)
	ependymoma	SIOP EPENDYMOMA II	NCT02265770	Will evaluate several molecular markers, including 1q copy numbers, Tenascin C, *RELA* fusions, *YAP* fusion, H3.3K27me3, and methylation
	hepatoblastoma and hepatocellular carcinoma	PHITT	NCT03017326	Develop genomic analysis to predict chemotherapy toxicity
	severe aplastic anaemia	EWOG-SAA-2010		Genetic characterisation study
	MDS or JNML	EWOG-MDS-2006		Genetic characterisation study
	any	OLCHC Tumour Bank		DNA biobanking
Multiple Types	multiple	MK7339-002 / LYNK-002	NCT03742895	Requires HRRm or HRD
	solid tumours	LOXO 101	NCT02576431	Requires *NTRK* fusion
	any	WAYFIND-R		Requires NGS tumour genomic profiling
	solid tumours	PUMA-NER 5201/SUMMIT	NCT01953926	Requires *HER2* mutation or *EGFR* exon 18 mutation
	cancer of unknown primary site	CUPISCO	NCT03498521	Will perform genomic profiling; excludes specific immunophenotypes

## Conclusions

Molecular diagnostics, in the form of both genetic and cellular biomarker testing, are a vital component of cancer diagnostics and treatment. In Ireland, the NCCP lists 148 treatment regimens with a molecular diagnostic component, through which 30% of the Irish cancer patient population stands to directly benefit. Cancer cases are predicted to double in Ireland by 2045 ^119^, underscoring the need to ensure that the increasing requirement for testing is met by Irish infrastructure. As research highlights further drug repurposing and new off-label drug uses, as novel precision medicine therapies are produced against innovative drug targets in more cancer types, and as clinical trials become more widely available in Ireland, the need for molecular testing is likely to increase steadily until the total number of required molecular tests converges with, and exceeds, the total number of cancer cases. It should also be noted that these numbers do not include testing for inherited cancer risk or any non-cancer disease, each of which will add to the requirement for molecular diagnostics. While this presents a challenge to any national healthcare system, it promises great improvements in personalised cancer care and outcomes for patients in the near future if the challenge can be met.

Ireland's recent National Genomics and Genetics Strategy will represent the first major strides in addressing this challenge. While the strategy encompasses many aspects, a key consideration that should be highlighted is the need for a collaborative approach from all stakeholders. Fundamental to this approach must be the facilitation of a modernised, centralised exchange of expertise and data from all parties, including the NCCP and NCRI for cancer expertise and statistics, the NCPE for pharmacoeconomics, hospitals for current infrastructure and implementation, and universities for current research efforts.

For this strategy to be successful, decisions must be based on accurate data gathered by these institutions. While genomics initiatives and strategies in countries with comparable population sizes (such as the Precision Medicine Centre of Excellence in Northern Ireland ^120^, the regional laboratories established through the Scottish Strategic Network for Genomic Medicine ^121^, the hub-and-spoke model employed in Denmark ^7^, or the distributed specialisation across institutions in Norway's InPreD initiative ^122^) can inform Irish efforts, it is critical to collect and analyse healthcare data in Ireland to establish a viable and appropriate molecular medicine service capable of meeting Irish clinical demand. This data will be foundational for evaluating the utility of clinical care in Ireland moving forward, particularly in pharmacoeconomic areas such as health technology assessments, pharmaceutical pricing, and drug reimbursement approvals. Furthermore, national infrastructure to support the collection and storage of molecular patient data will enable Ireland to participate in international research initiatives, such as the European Commission's Digital Europe Call for genomics data, and the proposed EU European Health Data Space ^123^.

In this article, we sought to collate available data from various sources across Ireland to present a unified overview of the state of cancer molecular diagnostics in Ireland. Ultimately, to best address Ireland's future need for molecular and genomic medicine, we first need to accurately establish Ireland's current capabilities and position, and it is our hope that others will follow in contributing to this

## Methods

### Molecular Diagnostics in Cancer Treatment Regimens

NCCP cancer therapy regimens were accessed via the HSE NCCP National SACT Regimens website ^15^. Information on therapy indications from each tumour group subpage (as well subpages for oral anti-cancer medicines and paediatric therapies) was collected by systematic HTML parsing of tabular elements using the Python package Beautiful Soup version 4.11.2 in Python 3.11.0 ^124^. Raw therapy indication text was then further parsed in Python to harmonise descriptions and drug names, to combine duplicate indications by indication ID, to assign relevant disease based on website subpage and subheadings, and to group therapy indications by regimen ID. Where conflicts arose in merging duplicate indications by ID, manual harmonisation was performed by referring to the full text of the hyperlinked regimen document; where conflicts arose in the hyperlinked regimen documents, the latest revision was used as reference. After tabular export of all indications and associated information, final manual curation was performed to correct malformed entries and errors in the source material, again referring to the appropriate full-text regimen documents. For the Python parser tool created for this purpose, see *Software Availability* ^125^ and for the exported and manually curated data table, see also *Software Availability* ^22^.

Identification of indications informed by genetic diagnostics was performed through several rounds of key-word search and manual review through the short descriptions of each therapy indication. Key-words included terms associated with genetics and genomics such as *gene*, *chromosome*, and *express*; known cancer gene names; and the terms and symbols *positive*, *negative*, *+*, and *-*, as well as further keywords encountered during manual review. In ambiguous cases, including cases where a molecular diagnostic was listed for one indication of a regimen, but not for other similar indications for the same regimen, both the full-text regimen document as well as published literature on the therapy in question were consulted. Note that while many regimens include CD20 antibody therapies for lymphoma, these were only included when a molecular diagnostic was explicitly referenced.

Reimbursement information was obtained from NCCP indications and regimens ^15^, the NCCP table of approved drugs ^23^, the PCRS list of reimbursable items ^24^, and the HSE list of the High Tech Drug Arrangements ^25^.

The regimen information in this article reflects the NCCP SACT Regimens website as of 2023-Nov-06.

### Predicted Rates of Actionable Cancer Molecular Diagnoses

Cancer incidence rates in Ireland were obtained from the NCRI publication *Cancer in Ireland 1994–2020: Annual Statistical Report of the National Cancer Registry*, *Appendix I: Incident Cancer Cases* ^46^, except where indicated. Case numbers in this publication are listed as the 3-year average incidence from 2018–2020 of each ICD-10 invasive cancer group.

For each unique molecular diagnostic for each cancer subtype, incidence of the cancer subtype relative to the broader cancer type (e.g., proportion of lung cancers that are NSCLC) was obtained from literature where appropriate. Incidence rates of each molecular diagnostic within the relevant cancer subtype (e.g., proportion of NSCLC that is *ALK*+) were then also obtained from literature (references provided in Table 3 and Table 4). These rates were then applied to incident cancer rates in Ireland to estimate the positivity rate of each molecular diagnostic in Ireland.

In the case of acute lymphoblastic leukaemia, B-ALL subtype incidence was estimated separately for paediatric and adult cases due to differences in B- vs T-ALL rates in adults and children and the high proportion of childhood cases ^72^. Similarly, separate molecular subtype rates were applied for *BCR-ABL1* fusions in adult and childhood B-ALL for the same reason ^73^. Incidence of metastatic castration-resistant prostate cancer was calculated as a function of total population based on the model referenced, producing numbers in agreement with NCRI case counts ^84, 85^. Rates of urothelial carcinoma were applied separately for primary urethral urothelial carcinoma due to lower published rates of urothelial histology ^105– 107^.

### Clinical Sequencing Capacity in Ireland

Technical specifications on the machine runtime and DNA throughput for the Illumina NextSeq and ThermoFisher Ion Torrent platforms were obtained from their respective manufacturer websites. To calculate nominal maximum throughput, the highest throughput configuration of each machine was used (NextSeq 550 High-Output = 100-120 Gb of 120 bp paired-end reads/29 hrs ^49^, Ion GeneStudio with Ion 550 Chip = 40-50 Gb of 200 bp paired-end reads per 12 hrs ^50^).

DNA sequencing target size was based on paired-end sequencing with a 120x coverage target using the Agilent SureSelect Clinical Research Exome V4 (total design size=51.0 Mb), for a targeted total of 12.24 Gb of genetic material sequenced per sample ^126^.

Maximum capacity was then calculated to be the total number of exomes able to be sequenced by all 8 machines running at maximum capacity. The lower end of this range represents one run of each instrument using the lower bound of the instruments' stated throughput (one 29-hour run of the NextSeq at 100 Gb = 8 exomes per machine = 32 exomes, plus one 12-hour run of the Ion Torrent at 40 Gb = 3 exomes per machine = 12 exomes, totalling 44 exomes), while the higher end of the range represents the higher bound of the instruments' stated throughput, with two 12-hour runs of the Ion Torrent within the same time frame as one 29-hour NextSeq run (one 29-hour run of the NextSeq at 120 Gb = 9 exomes per machine = 36 exomes, plus two 12-hour runs of the Ion Torrent at 50 Gb = 8 exomes twice per machine = 32 exomes, totalling 68 exomes).

### Molecular Indications for Clinical Trials

Information on clinical trials was obtained from Cancer Trials Ireland ^115^. Trials were considered to have a molecular component if the trial eligibility criteria included genetic mutations, aberrant genetic pathways, gene expression, cellular biomarkers, or microsatellite instability status as inclusion or exclusion criteria, or if the trial's purpose was to otherwise collect or analyse genomic data. Trials were evaluated systematically, beginning by prioritising those with explicit mention of these criteria in their short description. Trials without explicit reference to one of the two criteria, but which referenced a disease or treatment known to have a strong or common molecular diagnostic component were also prioritised. Short-listed trials' full trial descriptions were then checked to confirm the nature of the trial. After confirmation of short-listed trials, remaining trial full descriptions were then checked to confirm absence of a molecular diagnostic component.

## Data Availability

Zenodo: Table of Indications and Regimens from the National Cancer Control Programme, Ireland. https://zenodo.org/doi/10.5281/zenodo.10157939 ^22^ This project contains the following underlying data: NCCP_Indications_and_Regimens.2023-Nov-06.tsv Zenodo: Table of Indications and Regimens from the National Cancer Control Programme, Ireland. https://zenodo.org/doi/10.5281/zenodo.10157939 ^22^ This project contains the following extended data: nccp_sact_parser.py harmonization.tsv Data are available under the terms of the Creative Commons Zero "No rights reserved" data waiver (CC0 1.0 Public domain dedication). Analysis code available from: https://github.com/TDMedina/NCCP-SACT-Parser ^125^ Archived analysis code at time of publication: https://doi.org/10.5281/zenodo.10660553 License: Creative Commons Zero v1.0 Universal (CCO 1.0 Public domain dedication)
