# Supplementary material for: Overview of Molecular Diagnostics in Irish Clinical Oncology
Source: HRB Open Res. 2025 Jun 9;7:16. Originally published 2024 Mar 26. [Version 2] doi: 10.12688/hrbopenres.13822.2 (PMC12272048; doi:10.12688/hrbopenres.13822.2)
Supplement: Peer review history

# Reviewer report: Reviewer response for version 2

**DOI:** 10.21956/hrbopenres.15601.r47685

**Published:** 2025-07-17

**Authors:** George Thomas

**Affiliations:** 1 Department of Pathology & Laboratory Medicine, Knight Cancer Institute, Oregon Health & Science University, Portland, USA

This is fine,

 Thank you,

 G

Is the work clearly and accurately presented and does it cite the current literature?

Yes

If applicable, is the statistical analysis and its interpretation appropriate?

I cannot comment. A qualified statistician is required.

Are all the source data underlying the results available to ensure full reproducibility?

No source data required

Is the study design appropriate and is the work technically sound?

Yes

Are the conclusions drawn adequately supported by the results?

Yes

Are sufficient details of methods and analysis provided to allow replication by others?

Partly

Reviewer Expertise:

Precision Oncology;Molecular Diagnostics; Cancer Therapeutics: Companion Diagnostics; Pathology

I confirm that I have read this submission and believe that I have an appropriate level of expertise to confirm that it is of an acceptable scientific standard.

# Reviewer report: Reviewer response for version 1

**DOI:** 10.21956/hrbopenres.15129.r42367

**Published:** 2024-09-17

**Authors:** George Thomas

**Affiliations:** 1 Department of Pathology & Laboratory Medicine, Knight Cancer Institute, Oregon Health & Science University, Portland, USA

This study provides a comprehensive overview of molecular diagnostics in clinical oncology in Ireland. The authors analyze cancer treatment regimens published by the National Cancer Control Programme (NCCP), identifying 148 regimens with molecular diagnostic components. They estimate that approximately 50% of cancer patients in Ireland could qualify for molecular testing, with about 30% potentially benefiting from molecularly guided therapies. The paper examines the current sequencing capacity in Irish clinical laboratories and discusses the molecular criteria used in ongoing clinical trials. The study aims to establish a baseline understanding of current molecular diagnostic practices in Irish oncology and to inform future planning and implementation of personalized medicine approaches.

**Strengths:**

1. Comprehensive overview of molecular diagnostics in Irish clinical oncology, including genetic and biomarker testing.
2. Detailed analysis of NCCP cancer treatment regimens and associated molecular indications.
3. Estimation of molecular testing needs based on cancer incidence data and literature.
4. Assessment of current sequencing capacity in Irish clinical laboratories.
5. Inclusion of information on molecular criteria in ongoing clinical trials.

**Clarification and additional input needed:**

1. Limited discussion on the uniformity of testing across different centers in Ireland.
2. Minimal exploration of send-out testing practices and their implications.
3. Lack of detailed analysis on PD-L1 IHC testing and interpretation, despite its growing importance in oncology as a tumor agnostic biomarker for treatment.: Please specify the clones being used for testing, e.g. Dako 22C3
4. For NSCLC, the testing of RNA for ALK fusions should be described (ALK mutations suggest that this is not an RNA fusion event); also MET exon 14 skipping lesion, amplifications, etc.
5. It would help to separate Solid tumor from Hematological malignancies in the tables
6. GIST: Expand these to discuss whether in addition to c-KIT, testing also includes PDGFR, BRAF, NF1 and SDH complex
7. RAS mutations: please expand to include which genes, e.g. KRAS, NRAS, BRAF, etc. Substitute “normal RAS” with “ *Negative for RAS mutations”*
8. Melanoma: while BRAF V600E is most common, there are several other mutated codons that are present; also, NRAS and C-KIT mutations.
9. Need to expand on Her2 IHC testing results, i.e. Her-2 low; amplification, etc. and in different cancers
10. Expand on MSI testing, MLH1 methylation, BRAF mutations in the setting of colon cancer
11. Please define HRD+, i.e. what genes are considered?
12. Insufficient information on patient outcomes related to molecular testing.

**Additional areas that can be expanded on to strengthen this report:**

  

1. Analyze the uniformity of testing practices across different Irish hospitals and laboratories.
2. Explore the extent and implications of germline testing especially for breast, ovarian and prostate cancers
3. Incorporate data on patient outcomes related to molecular-guided therapies, if available.
4. Discuss challenges in standardizing molecular testing across different centers.
5. Examine the turnaround times for different tests and their impact on treatment decisions.

Is the work clearly and accurately presented and does it cite the current literature?

Yes

If applicable, is the statistical analysis and its interpretation appropriate?

I cannot comment. A qualified statistician is required.

Are all the source data underlying the results available to ensure full reproducibility?

No source data required

Is the study design appropriate and is the work technically sound?

Yes

Are the conclusions drawn adequately supported by the results?

Yes

Are sufficient details of methods and analysis provided to allow replication by others?

Partly

Reviewer Expertise:

Precision Oncology;Molecular Diagnostics; Cancer Therapeutics: Companion Diagnostics; Pathology

I confirm that I have read this submission and believe that I have an appropriate level of expertise to confirm that it is of an acceptable scientific standard, however I have significant reservations, as outlined above.

# Response

**Published:** 2025-06-04

**Authors:** Tyler Medina

**Affiliations:** University of Galway, Galway, County Galway, Ireland

Thank you, Prof. Thomas, for taking the time to review our manuscript and for providing valuable feedback. We have gone through your suggestions, and we have outlined our responses below. We would like to note that our manuscript was intended to provide an overview of the molecular markers that are described by the Irish National Cancer Control Panel for use in assigning cancer therapy, which limits our scope to the markers that have currently been described in drug regimens and indications by the NCCP. In our new release of the article, we have updated the described indications by reviewing and adding 98 indications that were added between November 2023 and May 2025.

 However, it should be noted that NCCP indications do not generally describe detailed testing strategies, which are instead described by the National Genomics Test Directory for Cancer, newly released and actively being developed since June 2024. We have now included additional diagnostic criteria for PD-L1 testing as suggested and as prescribed in the new national test directory. While there are also additional genetic tests outlined in the test directory (e.g., *DPYD *testing for fluoropyrimidine treatment, *BRAF *in mCRC, Lynch syndrome screening), any tests not yet included in NCCP drug regimens have not been added. In addition, Ireland does not as of today have a centralized electronic health record system, nor are centralized records kept regarding genomics tests ordered. These are matters of current development in the country. Similarly, because the National Genomics Test Directory was only very recently published, records on send-out practice have generally not been standardized or centralized, making them difficult to obtain even for the hospitals themselves.

**Comment 1: **Limited discussion on the uniformity of testing across different centers in Ireland.

**Response 1: **We were unfortunately unable to obtain records specifically detailing the molecular diagnostic practices at individual hospitals in Ireland and so we did not include a discussion on test practices across the country. However, Ireland has few hospitals performing cancer molecular diagnostics, with each generally specializing in particular testing or cancer types. For example, the majority of solid tumour samples in the country requiring NGS are sequenced at a single test center, while many breast cancer samples are sent for IHC at a separate center specializing in breast cancer. As such, testing is not so much uniform across hospitals as it is consolidated.

**Comment 2: **Minimal exploration of send-out testing practices and their implications.

**Response 2:** At the time of writing, no centralized records (or in-hospital records) regarding the number of send-out samples were available, and so these numbers could not be included in the article. Send-out practices were, in fact, an ongoing area of investigation by the new National Genetics and Genomics Office, and by several hospitals as they intended to start or expand NGS testing services. Domestic send-out, as mentioned in the response to point 1, is common, depending on the tissue type and testing required.

**Comment 3: **Lack of detailed analysis on PD-L1 IHC testing and interpretation, despite its growing importance in oncology as a tumor agnostic biomarker for treatment. Please specify the clones being used for testing, e.g. Dako 22C3

**Response 3: **Thank you for your comment. We had previously omitted required thresholds for PD-L1 expression for simplicity, but have added them in the revised version in Table 2. While specifications for antibodies were not previously available, the latest version of the National Genomics Test Directory for Cancer does now include this information for some tests for some cancer types, and we have added these to Table 2 where possible.

**Comment 4: **For NSCLC, the testing of RNA for ALK fusions should be described (ALK mutations suggest that this is not an RNA fusion event); also MET exon 14 skipping lesion, amplifications, etc.

**Response 4: **Thank you for pointing out the ambiguity in " *ALK *mutations". This has been corrected in the revised article in Tables 1 and 3 for NSCLC, with "mutation" replaced with "fusion". *MET *exon 14 was not included in the National Cancer Control Programme published list of drug regimens and indications when the original version of this article was released, but was added in July 2024, and it is now included in the article in Tables 1 and 3 for NSCLC as well.

**Comment 5: **It would help to separate Solid tumor from Hematological malignancies in the tables. 

**Response 5: **We have reordered the table entries in Tables 1 and 2 such that haematological malignancies (leukaemia/lymphoma) are at the bottom of each table. However, Tables 3 and 4 are grouped and sorted by incidence rates in Ireland, so we have elected to leave leukaemia and lymphoma in place to indicate their rank in total cancer cases.

**Comment 6: **GIST: Expand these to discuss whether in addition to c-KIT, testing also includes PDGFR, BRAF, NF1 and SDH complex

**Response 6: **At this time, NCCP guidelines on GIST testing for treatment are limited to *c-KIT*, and as such we have not included other genetic testing criteria, given the scope of the article, as outlined above.

**Comment 7: **RAS mutations: please expand to include which genes, e.g. KRAS, NRAS, BRAF, etc. Substitute “normal RAS” with “Negative for RAS mutations”

**Response 7: **Thank you for suggesting that this should be clarified. We have changed "normal *RAS*" to "wild-type *KRAS *and *NRAS*" for colorectal cancer in Tables 1 and 3 to avoid ambiguity and to be in line with the European Medicines Agency text regarding cetuximab and panitumumab indications.

**Comment 8: **Melanoma: while BRAF V600E is most common, there are several other mutated codons that are present; also, NRAS and C-KIT mutations.

**Response 8: **As of May 2025, *BRAF *V600 is the only codon currently listed under NCCP indications, and so other testing candidates are not listed. However, the national test directory as of April 2025 does now list testing for *BRAF*, *NRAS*, and *KIT*, with suggestions for test expansion to include *ALK *and *ROS*, and we anticipate that these genes will be added to NCCP indications if/when approved for targeted treatment.

**Comment 9: **Need to expand on HER2 IHC testing results, i.e. Her-2 low; amplification, etc. and in different cancers

**Response 9: **An indication for HER2-low in breast cancer was added to the NCCP indications in July 2024, and has now been added to the revised article (Tables 2 and 4). We have also further provided the distinguishing thresholds for HER2+ versus HER2-low in breast cancer in Table 2. However, a threshold for HER2+ in gastric cancers has not been officially published by the NCCP, and so is not included.

**Comment 10: **Expand on MSI testing, MLH1 methylation, BRAF mutations in the setting of colon cancer

**Response 10: **We have expanded on the genes indicated for dMMR testing (Table 1: endometrial and colorectal cancer) as per the national test directory, which now include *MLH1 *promoter hypermethylation, *PMS2*, *MSH2*, and *MSH6*. While *BRAF *is also included in the test directory for colon cancer, it is not yet included in any NCCP indication.

**Comment 11: **Please define HRD+, i.e. what genes are considered?

**Response 11: **The European Medicines Agency indication for the use of olaparib and bevacizumab in HRD+ gynaecological cancers bases its definition of HRD+ on the PAOLA-1 phase III clinical trial, which classified HRD+ patients as having either *BRCA1/2* tumour mutations or a sufficient genomic instability score (GIS). GIS was produced through the Myriad Genetics MyChoice CDx Plus assay, which considered factors such as genome-wide loss of heterozygosity, telomeric allelic imbalance, and large-scale transitions. The assay also includes 15 homologous repair pathway genes ( *ATM, BARD1, BRCA1, BRCA2, BRIP1, CDK12, CHEK1, CHEK2, FANCL, PALB2, PPP2R2A, RAD51B, RAD51C, RAD51D, *and *RAD54L*), though the assay and study both consider BRCA1/2 mutations alone to be sufficient for HRD+ classification in the study. Though the same Myriad assay has been considered for use in Ireland, the actual test implementation has not yet been officially established in the national test directory. However, as HRD+ and GIS refer to the EMA indication, we have now included their description of GIS as an explanatory note in the caption for Table 1, though we have not included the specific lists of genes used in the Myriad test, aside from *BRCA1/2*.

**Comment 12: **Insufficient information on patient outcomes related to molecular testing.

**Response 12: **This was a question that was initially considered as part of our investigation, as it would also greatly help contextualize the requirement for and benefit of molecular medicine in Ireland. Unfortunately, this data was not available to us as part of this study, but would be of great importance for future work.

**Additional areas that can be expanded on to strengthen this report:**

1. Analyze the uniformity of testing practices across different Irish hospitals and laboratories.
2. Explore the extent and implications of germline testing especially for breast, ovarian and prostate cancers
3. Incorporate data on patient outcomes related to molecular-guided therapies, if available.
4. Discuss challenges in standardizing molecular testing across different centers.
5. Examine the turnaround times for different tests and their impact on treatment decisions.

**Response:** Clinical implementation and results, including comparing testing practices, turnaround times, send-out practices, and patient outcomes, are certainly of interest to us, but were unfortunately out of scope for this current work. We expect that work regarding these matters will be more feasible in the coming years as national genomics initiatives in Ireland continue to progress and as data regarding these aspects of genomics are made more readily available.

# Reviewer report: Reviewer response for version 1

**DOI:** 10.21956/hrbopenres.15129.r41155

**Published:** 2024-07-15

**Authors:** Bálint Nagy

**Affiliations:** 1 Department of Human Genetics, Faculty of Medicine,, University of Debrecen, H-4032 Debrecen, Hungary

The submitted manuscripts is interesting and it is in the focus of clinical interest.

 The Abstract is too general, please provide more concrete data and conclusion. I miss liquid biopsy and cell-free nucleic acids, are  they in use in the clinical practice in Ireland? You should mention this possibility,  even if  it is not used in Ireland. The Methods section has to be more detailed. There is a "personalized medicine" keyword, you should discuss it in more details. Somehow you should give a perspective about the use of molecular genetics in the clinical practice, diagnosis,  treatment and follow up of the cases.

Is the work clearly and accurately presented and does it cite the current literature?

Yes

If applicable, is the statistical analysis and its interpretation appropriate?

Yes

Are all the source data underlying the results available to ensure full reproducibility?

Yes

Is the study design appropriate and is the work technically sound?

Yes

Are the conclusions drawn adequately supported by the results?

Partly

Are sufficient details of methods and analysis provided to allow replication by others?

Partly

Reviewer Expertise:

molecular genetics, molecular diagnostics, non.invasive diagnostics, cell-free nucleic acids, liquid biopsy, cancer

I confirm that I have read this submission and believe that I have an appropriate level of expertise to confirm that it is of an acceptable scientific standard.
